# Chronic activation of GPR40 does not negatively impact upon BRIN-BD11 pancreatic β-cell physiology and function

**DOI:** 10.1007/s43440-020-00101-6

**Published:** 2020-04-09

**Authors:** Eloisa Aparecida Vilas-Boas, Noémie Karabacz, Gabriela Nunes Marsiglio-Librais, Maíra Melo Rezende Valle, Lisa Nalbach, Emmanuel Ampofo, Bruce Morgan, Angelo Rafael Carpinelli, Leticia Prates Roma

**Affiliations:** 1 Department of Physiology and Biophysics, Institute of Biomedical Sciences, University of Sao Paulo (USP), Sao Paulo, SP Brazil; 2 Department of Biophysics, Center for Human and Molecular Biology, Saarland University, Universität Des Saarlandes, CIPMM, Geb. 48, 66421 Homburg/Saar, Germany; 3 Institute for Clinical and Experimental Surgery, Saarland University, 66421 Homburg/Saar, Germany; 4 Institute of Biochemistry, Center for Human and Molecular Biology (ZHMB), Saarland University, 66123 Saarbrücken, Germany

**Keywords:** Lipotoxicity, GPR40 activation, β-cells, Palmitate

## Abstract

**Background:**

Free fatty acids (FFAs) are known for their dual effects on insulin secretion and pancreatic β-cell survival. Short-term exposure to FFAs, such as palmitate, increases insulin secretion. On the contrary, long-term exposure to saturated FFAs results in decreased insulin secretion, as well as triggering oxidative stress and endoplasmic reticulum (ER) stress, culminating in cell death. The effects of FFAs can be mediated either via their intracellular oxidation and consequent effects on cellular metabolism or via activation of the membrane receptor GPR40. Both pathways are likely to be activated upon both short- and long-term exposure to FFAs. However, the precise role of GPR40 in β-cell physiology, especially upon chronic exposure to FFAs, remains unclear.

**Methods:**

We used the GPR40 agonist (GW9508) and antagonist (GW1100) to investigate the impact of chronically modulating GPR40 activity on BRIN-BD11 pancreatic β-cells physiology and function.

**Results:**

We observed that chronic activation of GPR40 did not lead to increased apoptosis, and both proliferation and glucose-induced calcium entry were unchanged compared to control conditions. We also observed no increase in H_2_O_2_ or superoxide levels and no increase in the ER stress markers p-eIF2α, CHOP and BIP. As expected, palmitate led to increased H_2_O_2_ levels, decreased cell viability and proliferation, as well as decreased metabolism and calcium entry. These changes were not counteracted by the co-treatment of palmitate-exposed cells with the GPR40 antagonist GW1100.

**Conclusions:**

Chronic activation of GPR40 using GW9508 does not negatively impact upon BRIN-BD11 pancreatic β-cells physiology and function. The GPR40 antagonist GW1100 does not protect against the deleterious effects of chronic palmitate exposure. We conclude that GPR40 is probably not involved in mediating the toxicity associated with chronic palmitate exposure.

**Electronic supplementary material:**

The online version of this article (10.1007/s43440-020-00101-6) contains supplementary material, which is available to authorized users.

## Introduction

Free fatty acids (FFAs) play a central role in cellular physiology as they are both components of biological membranes and an important cellular energy source. In pancreatic β-cells, FFAs can acutely enhance glucose-stimulated insulin secretion (GSIS) either by modulation of intracellular metabolism or via activation of specific G protein-coupled receptors (GPCRs) [1–4]. In contrast, chronic exposure of pancreatic β-cells to saturated FFAs leads to impaired insulin secretion, β-cell dysfunction and apoptosis, a condition that has been termed lipotoxicity [5–12].

Lipotoxicity is a complex condition and several different molecular mechanisms have been suggested to underlie chronic FFA toxicity in β-cells. These mechanisms include, (i) generation of reactive oxygen species (ROS) from both mitochondrial and non-mitochondrial sources, accompanied by a reduced expression of antioxidant enzymes [10, 12, 13]; (ii) induction of ER stress, particularly the unfolded protein response (UPR) [6–9, 14]; and (iii) chronic stimulation of the FFA receptor GPR40 (FFAR1), which ultimately leads to apoptosis [15–17].

**Fig. 1:**
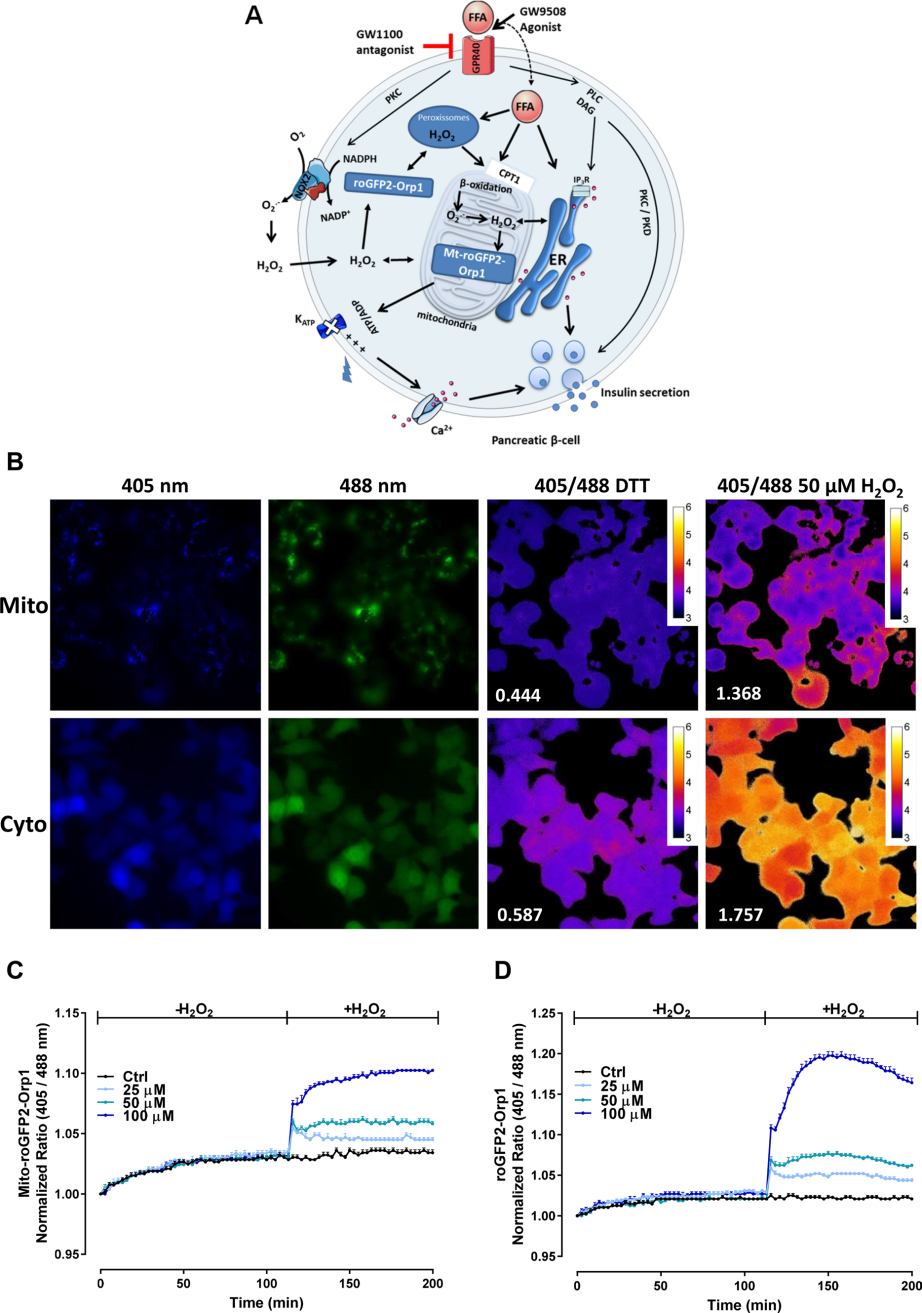
Signaling pathways activated by FFA and GPR40 agonists. **a** FFAs can enter the cells and be translocated to the mitochondria via carnitine palmitoyltransferase 1 (CPT1) to be oxidized by β-oxidation. Long- and very-long-chain fatty acids are oxidized in peroxisomes, which generates H_2_O_2_. Long-chain saturated or unsaturated FFAs can also bind to Gαq-protein-coupled receptor GPR40 located in the plasma membrane. Acute activation of GPR40 by FFAs or agonists as GW9508 activates the phospholipase C (PLC) / diacylglycerol (DAG) pathway, which, respectively, activates PKC and mobilizes calcium from the endoplasmic reticulum (ER), potentiating the insulin release. GPR40 activation also activates PKD1, which acts on actin filaments to increase insulin secretion. Both activation of PKC and the β-oxidation may lead to the production of reactive oxygen species (ROS) in the cytosol by the NADPH oxidase 2 (NOX2) complexes and by the electron transport chain in the mitochondrial matrix, respectively. Specific sensors for hydrogen peroxide (H_2_O_2_) targeted to the mitochondrial matrix (Mt-roGFP2-Orp1) or in the cytosol (roGFP2-Orp1) were used herein to assess the H_2_O_2_ production by different compartments. **b** Fluorescence of BRIN-BD11 cells expressing roGFP2-Orp1 in the mitochondrial matrix (Mito) or in the cytosol (Cyto) following excitation at 405 nm or 488 nm and the ratio (405/488) after addition of 10-mM DTT or 50-µM H_2_O_2_. Numbers represent the non-normalized ratio after addition of DTT or 50 µM H_2_O_2_. **c**, **d** Response of Mt-roGFP2-Orp1 (**c**) or roGFP2-Orp1 cells (**d**) expressed in BRIN-BD11 exposed to exogenously applied H_2_O_2_ at the concentrations indicated

GPR40 (FFAR1) is a GPCR that is activated by both saturated and unsaturated, medium- to long-chain FFAs, including palmitate, oleate and linoleate [18–20]. Previously, it was reported that GPR40, which is highly expressed in pancreatic β-cells, contributes to FFA-induced GSIS [21]. Further studies showed that the Gαq-PLC pathway, PDK-1, and intracellular calcium are likely involved in this effect [4, 20–22] (Fig. 1a). Based in part on these results, GPR40 emerged as a promising therapeutic target for type 2 diabetes mellitus (T2DM), resulting in the development of several agonist molecules targeting this receptor [23–26].

Positive effects of GPR40 agonists on insulin secretion and viability have been reported in studies employing both rodent and human-derived pancreatic islets, as well as in β-cell lines (reviewed elsewhere) [27, 28]. Furthermore, the GPR40 agonist, TAK-875, reached the stage for human clinical trials, demonstrating improved insulin secretion and little risk for hypoglycemic events [25, 29–32]. In contrast, other studies have reported that chronic stimulation of GPR40 may be related to impaired insulin secretion and apoptosis [15, 17, 33–35] and the clinical trial using TAK-875 was terminated prematurely due to problems with liver toxicity [36]. Therefore, to date, no consensus has been reached concerning the impact of chronic GPR40 stimulation on β-cell function and fitness. Potential molecular mechanisms relating chronic GPR40 stimulation to changes in β-cell physiology are, thus, likewise very unclear. Consequently, the safety and efficacy of long-term use of GPR40 agonists in human patients remain an open question.

In this study, we sought to investigate the impact of chronic GPR40 activation on β-cell viability and function using the GPR40 agonist GW9508. Specifically, we focused on Ca^2+^ homeostasis, induction of UPR and changes in intracellular H_2_O_2_ levels. Furthermore, we employed a GPR40 antagonist (GW1100) to investigate the role of GPR40 in the deleterious effects of chronic palmitate exposure. We observed that chronic GPR40 stimulation does not lead to changes in pancreatic β-cell viability, Ca^2+^ homeostasis, UPR induction or H_2_O_2_ levels. Furthermore, we observed no protective effect of GRP40 inhibition in chronic palmitate exposure.

## Results

### Chronic activation of GPR40 signaling does not lead to increased H_2_O_2_ production

Acute GPR40 activation has been shown to lead to increased ROS production via NADPH oxidases. However, the impact of chronic GPR40 activation on cellular ROS production is unclear. We considered it plausible that a long-term stimulation of ROS production may be involved in the reported toxicity of chronic GPR40 activation by small molecule GPR40 agonists.

Many methods for measuring intracellular ROS are prone to artifacts, are not able to monitor dynamic changes in ROS levels, offer little insight into subcellular ROS distribution and have poor ROS species specificity. We, therefore, sought to use the genetically encoded H_2_O_2_ sensor, roGFP2-Orp1, targeted to either the mitochondrial matrix or the cytosol/nucleus to overcome some of these limitations and to gain insight into subcellular compartment-specific H_2_O_2_ changes following chronic GPR40 activation [37, 38]. Specifically, we generated BRIN-BD11 cells stably expressing the mitochondrial matrix-targeted H_2_O_2_ sensor, mito-roGFP2-Orp1, or the cytosolic/nuclear localized sensor, roGFP2-Orp1. First, we demonstrated successful expression of the mitochondrial and cytosolic/nuclear sensor in BRIN-BD11 cells following excitation at either 405 nm or 488 nm (Fig. 1b). Fluorescence microscopy revealed that both sensors respond as expected to disulfide reducing (10-mM DTT) and oxidizing (50-μM H_2_O_2_) conditions, as indicated by ratiometric changes in fluorescence emission intensity following excitation at either 405 nm or 488 nm, henceforth referred to as the 405/488 nm ratio (Fig. 1b).

Next, we developed a fluorescence plate reader-based assay to enable us to monitor dynamic changes in roGFP2-Orp1 oxidation and, thus, H_2_O_2_ levels. With this assay, we observed a dose-dependent increase in the 405/488 nm ratio and, therefore, in roGFP2-Orp1 oxidation in both the mitochondrial matrix (Fig. 1c) and the cytosol (Fig. 1d), following the exogenous addition of H_2_O_2_. We, therefore, conclude that both sensors are capable of reporting changes in H_2_O_2_ concentrations in BRIN-BD11 cells.

**Fig. 2:**
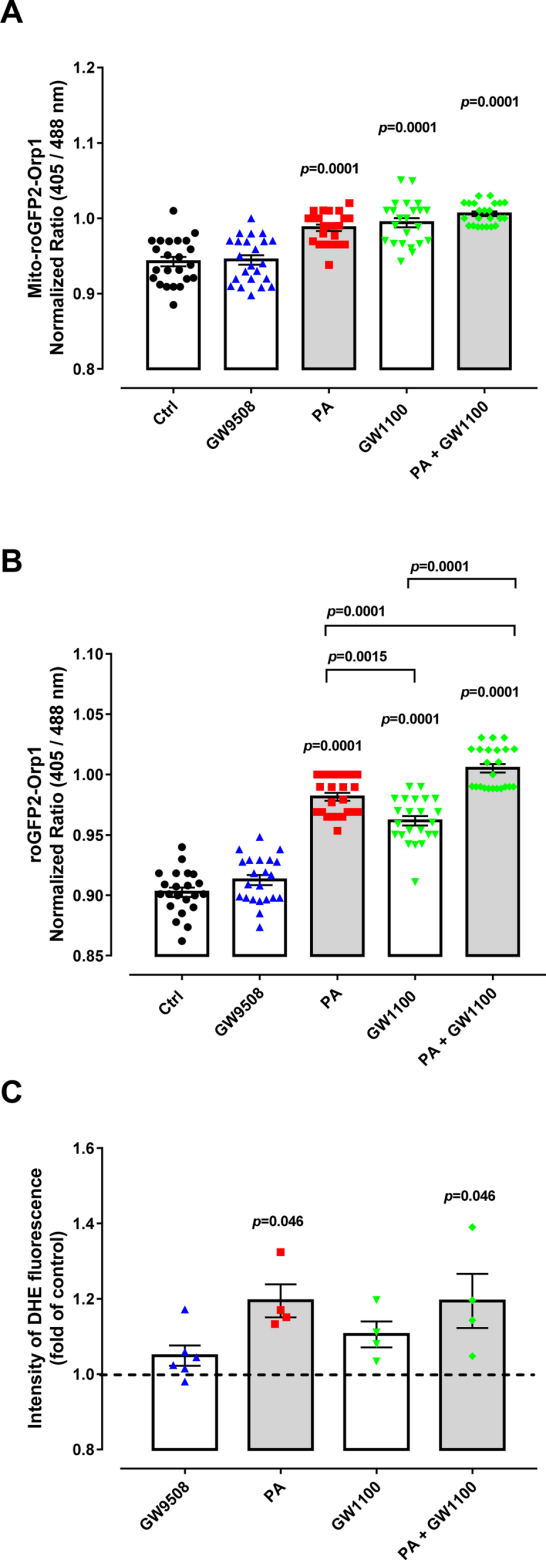
ROS production of BRIN-BD11 cells. **a**, **b** Fluorescence excitation ratio of Mt-roGFP2-Orp1 (**a**) or roGFP2-Orp1 (**b**) expressed in BRIN-BD11 cells after 48 h treatment with either: control (Ctrl), 100 µM Palmitate (PA), 20 μM GPR40 agonist (GW9508), 10 μM GPR40 antagonist (GW1100) and PA + GW1100 in culture media with 10 mM glucose. Results are expressed as the normalized ratio (405/488 nm) against time (mean ± SEM) of 4–6 technical replicates from four independent experiments. Two-way ANOVA followed by Tukey was used to compare Ctrl, PA, GW1100 and PA + GW1100; Student’s *t* test was used to compare Ctrl and GW9508. In (**a**) there was a significant effect of PA (*F*_1,88_ = 31.06, *p* < 0.0001) and GW1100 (*F*_1,88_ = 47.59, *p* < 0.0001). In (**b**) there was a significant effect of PA (*F*_1,87_ = 279.7, *p* < 0.0001) and GW1100 (*F*_1,87_ = 129, *p* < 0.0001). *p* values are shown on the graph and indicate the difference versus control or difference versus PA in brackets. **c** Superoxide content after 48 h of incubation was analyzed by flow cytometry of cells treated with DHE. Conditions: control (Ctrl), 100-µM palmitate (PA), 20-μM GPR40 agonist GW9508 (GW9508), 10-μM GPR40 antagonist (GW1100), PA + GW1100 in culture media with 10-mM glucose for 48 h. Results are expressed as mean ± SEM for 4–6 independent experiments. Two-way ANOVA followed by Tukey was used to compare Ctrl, PA, GW1100 and PA + GW1100; Student’s t test was used to compare Ctrl and GW9508. There was a significant effect of PA (*F*_1,12_ = 9.672, *p* = 0.0090) and not GW1100 (*F*_1,12_ = 1.335, *p* = 0.2703). *p* values are shown at the graph and indicate the difference versus control

We next investigated whether chronic treatment of our sensor-expressing BRIN-BD11 cells, with the GPR40 agonist GW9508, leads to H_2_O_2_ changes. GW9508 is an agonist of the FFAs receptors GPR40 and GPR120, with 100-fold selectivity to GPR40 in comparison to GPR120 [19]. To assess H_2_O_2_ changes, we incubated BRIN-BD11 cells for 48 h with GW9508 and subsequently measured the 405/488 nm ratio of both mitochondrial matrix (Fig. 2a) and cytosol (Fig. 2b) targeted roGFP2-Orp1 sensors. Interestingly, in both compartments, we observed no change in roGFP2 oxidation and, therefore, no change in H_2_O_2_ levels. Furthermore, we incubated cells with the superoxide (O_2_^.−^)-reactive small molecule dye, dihydroethidium (DHE). Similarly, we also observed no increase in DHE fluorescence in GW9508-treated cells (Fig. 2c). In contrast, chronic treatment with the GPR40 non-competitive antagonist, GW1100, led to significantly increased oxidation of the mitochondrial matrix (Fig. 2a) and cytosolic (Fig. 2b) roGFP2-Orp1 sensors, as well an apparent increase in DHE fluorescence, although this was not statistically significant (Fig. 2c).

Acute palmitate exposure has been shown to increase ROS production both via activation of NADPH oxidases and mitochondrial respiration [2, 39–41] as well as by GPR40 activation [1–4]. Chronic palmitate exposure also leads to increased ROS [42, 43]; however, whether GPR40 is involved in this context is unclear. We were able to recapitulate the previously reported increased in ROS production upon chronic palmitate exposure. Specifically, after 48 h of palmitate treatment, we observed an increased oxidation of both mitochondrial matrix (Fig. 2a) and cytosolic (Fig. 2b) roGFP2-Orp1 sensors, as well as increased DHE fluorescence (Fig. 2c), supporting an increase in O_2_^.−^ production and subsequent dismutation to H_2_O_2_.

Finally, we monitored cells treated with both GW1100 and palmitate in combination to test for any involvement of GPR40 in ROS production upon chronic palmitate treatment. For the mitochondrial matrix roGFP2-Orp1 sensor, we observed a similar increase in oxidation as for palmitate or GW1100 treatments alone (Fig. 2a). For the cytosolic roGFP2-Orp1 sensor, we observed an increase in oxidation larger than for that with palmitate or GW1100 treatments alone (Fig. 2b). With DHE we also observed a statistically significant increase in fluorescence emission compared to control (Fig. 2c). In conclusion, our results surprisingly indicate that chronic GPR40 inhibition, and not chronic activation, leads to increased O_2_^.−^/H_2_O_2_ levels. Hence, we suggest that GPR40 activation is not involved in chronic palmitate exposure-induced O_2_^.−^/H_2_O_2_ production.

### Chronic GPR40 activation or inhibition has no effect on cell viability

**Fig. 3:**
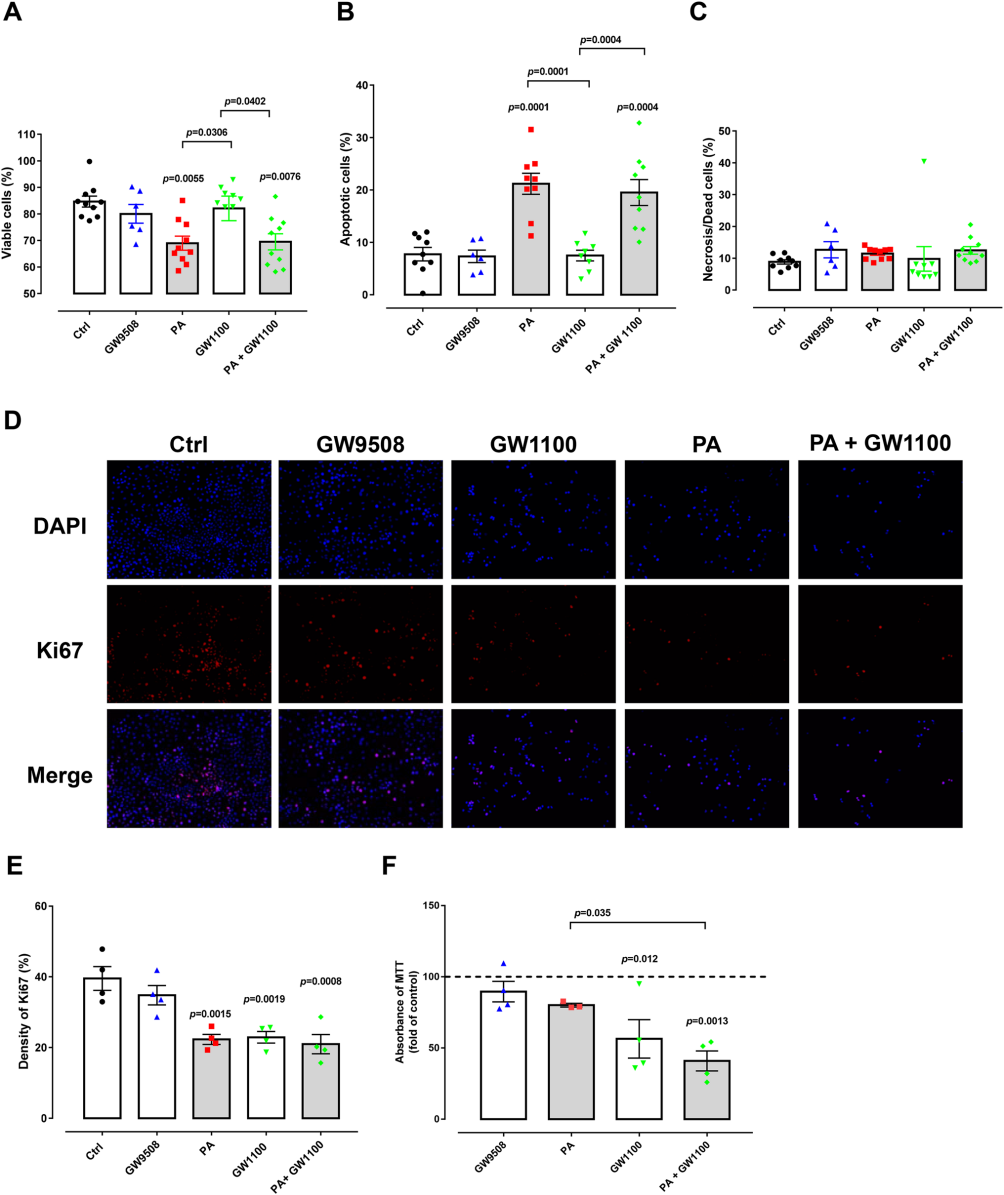
Apoptosis, metabolic activity and proliferation of BRIN-BD11 cells. Cells were divided into: control condition (Ctrl), 100-µM palmitate (PA), 20-μM GPR40 agonist (GW9508), 10 μM GPR40 antagonist (GW1100), PA + GW1100 and were treated in 10-mM glucose for 48 h. Percentage of viable (**a**), apoptotic (**b**) and necrotic (**c**) BRIN-BD11 cells after treatment in the different conditions. Analysis was performed by flow cytometry using the ViaCount reagent. Results are expressed as mean ± SEM for 6–10 independent experiments. Two-way ANOVA followed by Tukey was used to compare Ctrl, PA, GW1100 and PA + GW1100; Student’s *t* test was used to compare Ctrl and GW9508. There was a significant effect of PA (*F*_1,31_ = 48.2, *p* < 0.0001) and not GW1100 (*F*_1,31_ = 0.2766, *p* = 0.6027). *p* values are shown at the graph and indicate the difference versus control or difference versus PA in brackets. **d** DAPI and Ki67 staining of BRIN-BD11 cells after treatment in different conditions. **e** Density of Ki67 (%). Results are expressed as mean ± SEM for four independent experiments. Two-way ANOVA followed by Tukey was used to compare Ctrl, PA, GW1100 and PA + GW1100; Student’s *t* test was used to compare Ctrl and GW9508. There was a significant effect of PA (*F*_1,12_ = 15.64, *p* = 0.0019) and GW1100 (*F*_1,12_ = 13.75, *p* = 0.0030). *p* values are shown at the graph and indicate the difference versus control. Images were quantified at ImageJ Fiji Software. **f** MTT assay of BRIN-BD11 cells after treatment with the different conditions as detailed above. Absorbance was measured at 570 nm. Results are expressed as mean ± SEM for four independent experiments. Two-way ANOVA followed by Tukey was used to compare Ctrl, PA, GW1100 and PA + GW1100; Student’s t test was used to compare Ctrl and GW9508. There was a significant effect of GW1100 (*F*_1,11_ = 24.92, *p* = 0.0004) and not PA (*F*_1,11_ = 4.555, *p* = 0.0562). *p* values are shown at the graph and indicate the difference versus control or difference versus PA in brackets

We next tested whether chronic GPR40 activation or inhibition affects cell viability. We, therefore, treated BRIN-BD11 cells with GW9508 or GW1100 for 48 h and subsequently subjected the cells to three different assays to assess viability and proliferation. Using a flow cytometric-based assay for apoptotic cells (see materials and methods), we observed no decrease in the percentage of viable cells and no increase in the percentage of apoptotic or necrotic cells with either chronic GW9508 or GW1100 treatment (Fig. 3a–c). Likewise, immunostaining against Ki67, a marker of cellular proliferation, revealed no impact of GW9508 treatment (Fig. 3d, e). However, we did see a decrease in proliferation of cells chronically exposed to GW1100 (Fig. 3d, e). Finally, consistent with Ki67 staining data, a 3-[4,5-dimethylthiazole-2-yl]-2,5-diphenyltetrazolium bromide (MTT)-based assay for cellular metabolic activity revealed no impact of chronic GW9508 treatment (Fig. 3f), but did show decreased metabolic activity following chronic GW1100 exposure (Fig. 3f).

We tested whether GPR40 has any role in the toxicity of chronic palmitate exposure on β-cell viability. We observed a strong and significant decrease in the number of viable cells and an increase in the number of apoptotic cells following chronic palmitate treatment (Fig. 3a, b), with no changes in the number of necrotic cells (Fig. 3c). However, co-treatment of cells with GW1100 had no effect on palmitate-induced apoptosis (Fig. 3b). Furthermore, we observed that both palmitate and GW1100, either alone or in combination, led to decreased proliferation (Fig. 3d, e) and decreased metabolic activity (Fig. 3f). Therefore, we conclude that palmitate-induced β-cell apoptosis is probably not mediated through GPR40 activation. On the contrary, GPR40 inhibition appears to be detrimental to β-cell metabolic activity and proliferation, although the negative effects of GPR40 inhibition and palmitate treatment do not appear to be additive.

### GPR40 is not involved in the calcium response of BRIN-BD11 cells to glucose

We were next interested to assess whether chronic GPR40 stimulation or inhibition affects the ability of BRIN-BD11 cells to respond to acute glucose addition. Increase in intracellular Ca^2+^ upon glucose stimulation is directly involved in the mobilization and secretion of the insulin granules, thereby reflecting β-cell functionality. Therefore, we incubated BRIN-BD11 cells for 48 h with GW9508, GW1100, palmitate or palmitate in combination with GW1100. Subsequently, we used the Ca^2+^ dye, Fura-2 AM, to monitor cytosolic Ca^2+^ changes in response to acute addition of 20-mM glucose.

**Fig. 4:**
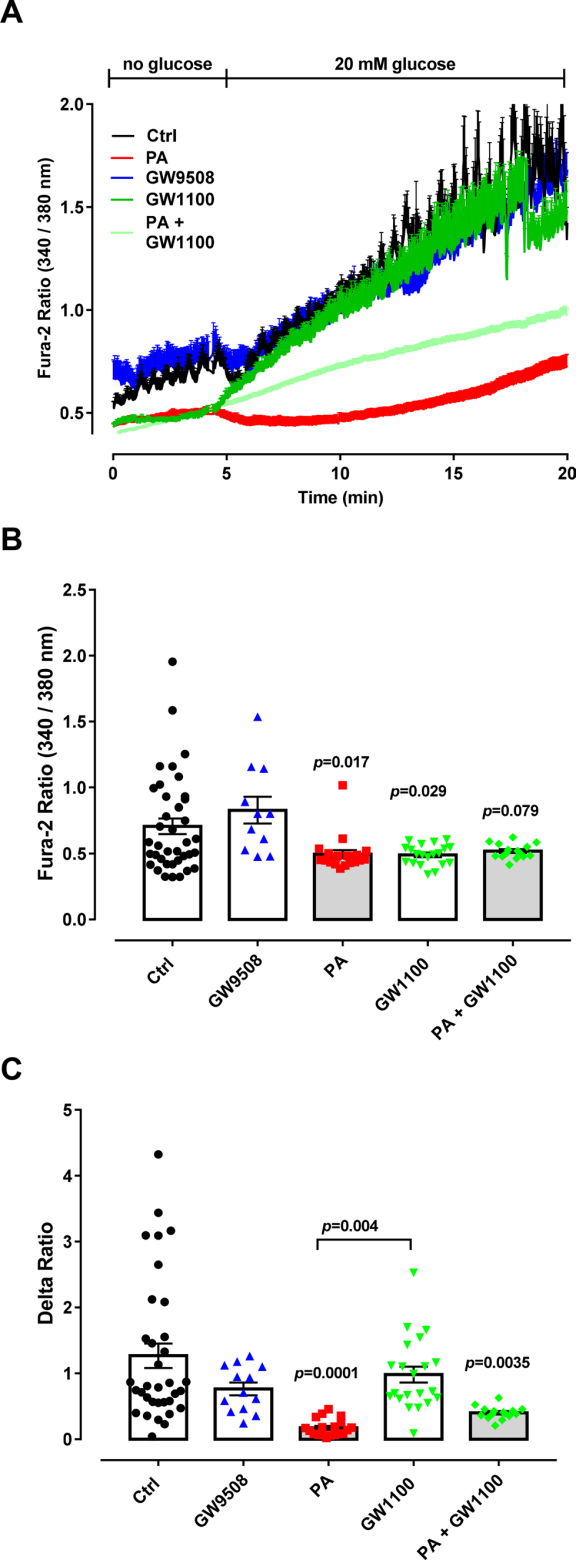
Dynamic measurements of Ca^2+^ in BRIN-BD11 cells. Cells were divided into: control condition (Ctrl), 100-µM palmitate (PA), 20-μM GPR40 agonist(GW9508), 10-μM GPR40 antagonist (GW1100) and PA + GW1100, treated in 10-mM glucose for 48 h. After the incubation, Ca^2+^ measurements were performed using Fura-2 AM dye using an Axio Observer seven microscope. Cells were first incubated for 5 min without glucose followed by up to 20 min with 20 mM of glucose. **a** Calcium dynamics. Glucose concentration is indicated above. Two-way ANOVA followed by Tukey was used to compare Ctrl, PA, GW1100 and PA + GW1100; Student’s *t* test was used to compare Ctrl and GW9508. **b** Resting Ca^2+^ was calculated as an average of the first 5 min of measurement before glucose addition. There was no significant effect of PA (*F*_1,90_ = 3.423, *p* = 0.0676) or GW1100 (*F*_1,90_ = 2.399, *p* = 0.1250). **c** Delta was calculated by averaging data at 18 min minus the average of data at 4 min. There was a significant effect of PA (*F*_1,87_ = 24.89, *p* < 0.0001) and not GW1100 (*F*_1,87_ = 0.0001879, *p* = 0.9891). Results are expressed as mean ± SEM for three independent experiments. *p* values are shown on the graph and indicate the difference versus control

We analyzed two parameters: the resting Ca^2+^ after culture in different conditions, which was calculated by averaging Ca^2+^ in the first 5 min of measurement before the addition of glucose (Fig. 4b), and, the change in the Fura-2 AM fluorescence excitation ratio following 14 min of acute glucose treatment (Fig. 4c). Interestingly, GW1100, but not GW9508, led to a decrease in resting Ca^2+^ levels. Palmitate treatment also led to a statistically significant decrease in resting Ca^2+^ levels (Fig. 4a, b). However, chronic treatment with either GW9508 or GW1100 did not affect the ability of cytosolic Ca^2+^ to respond to acute glucose treatment (Fig. 4a, c). In contrast, chronic treatment with palmitate abolished the cytosolic Ca^2+^ response to acute glucose treatment (Fig. 4c). Co-treatment of cells with GW1100 and palmitate did not rescue the negative effects of palmitate on either resting Ca^2+^ levels or the ability of Ca^2+^ to respond to glucose (Fig. 4a–c). In summary, we conclude that chronic GPR40 activation or inhibition does not affect the ability of BRIN-BD11 cells to respond to acute glucose treatment.

### Chronic GPR40 activation or inhibition does not lead to ER stress

**Fig. 5:**
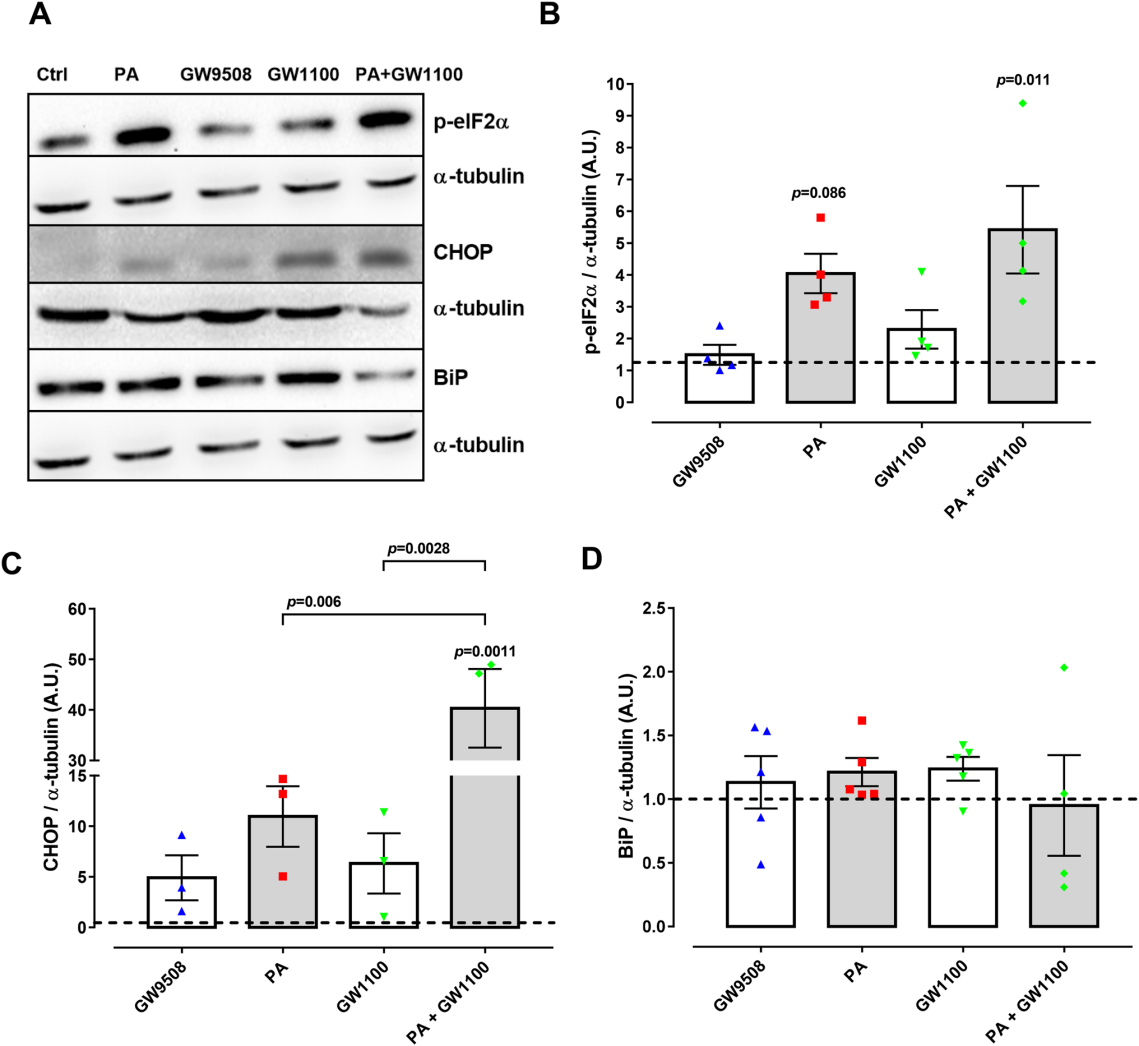
Expression of ER stress markers in BRIN-BD11 cells. Cells were divided into: control condition (Ctrl), 100-µM palmitate (PA), 20-μM GPR40 agonist GW9508 (GW9508), 10 μM GPR40 antagonist (GW1100), PA + GW1100 and were treated in media containing 10-mM glucose for 48 h. Western blot analysis was performed for the proteins of interest: p-eIF2α, CHOP and BiP, using α-tubulin as internal control. **a** Representative bands and respective α-tubulin. Densitometric analysis of western blot: p-eIF2α (**b**), CHOP (**c**) and BiP (**d**). Results are expressed as mean ± SEM of 3–5 independent experiments. Two-way ANOVA followed by Tukey was used to compare Ctrl, PA, GW1100 and PA + GW1100; Student’s *t* test was used to compare Ctrl and GW9508. **b** There was a significant effect of PA (*F*_1,12_ = 14.41, *p* = 0.0025) and not GW1100 (*F*_1,12_ = 2.691, *p* = 0.1269). **c** There was a significant effect of PA (*F*_1,8_ = 24.68, *p* = 0.0011) and GW1100 (*F*_1,8_ = 15.35, *p* = 0.0044). **d** There was no significant effect of PA (*F*_1,15_ = 0.04331, *p* = 0.8379) or GW1100 (*F*_1,15_ = 0.004461, *p* = 0.9476). *p* values are shown on the graph and indicate the difference versus control or difference versus PA in brackets

It is well understood that chronic exposure to palmitate leads to ER stress, which induces UPR activation [8]. Finally, therefore, we tested whether GPR40 plays a role in mediating the induction of ER stress. To this end, we monitored by western blot the expression of three key UPR-regulated proteins, namely p-eIF2α, BiP and CHOP after 48 h treatment of BRIN-BD11 cells with GW9508, GW1100, palmitate or palmitate and GW1100 in combination. We observed no effect of GW9508 or GW1100 on p-eIF2α or CHOP levels (Fig. 5a–c). In contrast, palmitate tends to increase both p-eIF2α and CHOP levels, which was not rescued in cells co-treated with GW1100 (Fig. 5a–c). Finally, we observed that no treatment had any effect on BiP levels (Fig. 5a, d). However, it is well known that BiP responds to chronic palmitate treatment at much earlier time-points and that BiP levels have been recovered by 48 h [8]. In conclusion, chronic GPR40 activation or inhibition does not induce ER stress.

## Discussion

T2DM is a serious and growing world health problem. Therefore, there is a high degree of interest in the development of novel drugs aimed at improving pancreatic β-cell function and survival, decelerating disease progression and enhancing patient well-being.

GPR40 is a highly expressed FFA receptor that has been shown to promote insulin secretion in pancreatic β-cells in response to both FFAs and glucose [27]. Consequently, there has been considerable interest in the development of GPR40 agonists as a possible therapeutic agent for stimulating pancreatic β-cells and, thus, for treatment of T2DM. Several GPR40 agonists have demonstrated positive effects on glycemic control in rodents and humans [27, 28]. Nonetheless, some studies have indicated that chronic activation of GPR40 may be detrimental for β-cell fitness and therefore, on the contrary, GPR40 antagonists may be beneficial for T2DM treatment [15–17, 33–35]. Finally, there is considerable discussion concerning the role of GPR40 in fatty acid-induced β-cell toxicity [17, 44].

In this study, we able to gain further insight into the impact of agonist and antagonist-based chronic activation and inhibition of GPR40 on BRIN-BD11 pancreatic β-cell line function and viability, as well as the role of GPR40 in mediating the effects of chronic palmitate exposure. Principally, we found that agonist-based chronic activation of GPR40, did not lead to increased cell apoptosis nor did it lead to decreased cellular proliferation or loss of metabolic activity. Furthermore, it did not affect Ca^2+^ homeostasis or the response of Ca^2+^ to glucose and did not lead to increases in cytosolic or mitochondrial H_2_O_2_ nor to increased superoxide levels. Therefore, our data suggest that 48 h activation of GPR40 does not lead to cellular toxicity in BRIN-BD11 β-cells. Our results, therefore, do not support a negative role of chronic GPR40 activation on β-cells. In agreement, Wagner and colleagues have also shown that cells treated with another synthetic GPR40 agonist, TUG-469, did not show any indication of increased apoptosis. Furthermore, when incubated with PA, the GPR40 agonist TUG-469, protected INS-1E cells from apoptosis [45]. Although toxic effects of the GPR40 agonist TAK-875 on hepatic cells have been reported during phase III clinical trials, it is currently unclear if toxicity was confined to hepatic cells or affected other cells as well [36].

Interestingly, we show that chronic incubation of BRIN-BD11 cells with a GPR40 antagonist, GW1100, led to decreased cellular proliferation and metabolic activity. Furthermore, 48 h exposure to the antagonist also increased cytosolic and mitochondrial matrix H_2_O_2_ levels. Based on our data, we hypothesize that decreased cellular metabolism (and consequently NADPH levels, as suggested by MTT assay) could underlie the effects of GW1100 treatment on H_2_O_2_ production. This is plausible as NADPH is the ultimate source of reductive equivalents for many H_2_O_2_-scavenging enzymes. Therefore, the lack of NADPH might ultimately be responsible for increased H_2_O_2_ levels, as previously shown in pancreatic islets cultured with low glucose [46, 47]. These data are also in agreement with the toxic effects of another GPR40 antagonist TUG-761. When INS-1E cells were incubated with TUG-761, it led to increased apoptosis, which was not further augmented with co-incubation of palmitate [45].

It has recently been shown, using several different antagonists (ANT203, DC260126 or ANT825) as well as by GPR40 knockdown, that the GPR40 pathway mediates a palmitate-induced increase in mitochondrial respiration in Min6 cells and human islets [48]. The mechanism by which inhibition of GPR40 impacts upon respiration is not completely clear, but might involve PKC, Ca^2+^ and production of signaling second messengers [48]. Here, we have also shown decreased metabolism upon GPR40 antagonism as well as lower resting Ca^2+^ (Figs. 3f, 4b, respectively). Those changes could consequently directly impact on proliferation rate, as, for example, seen by decreased Ki67 staining in GW1100-treated cells (Fig. 3d, e). Finally, treatment with a GPR40 antagonist did not ameliorate any of the negative effects of chronic palmitate treatment and, therefore, in our cells, palmitate-induced toxicity is likely to be independent of GPR40 signaling.

In contrast to our results, several recent studies have suggested that antagonism of GPR40 may represent a more effective method for T2DM treatment as compared to GPR40 agonism [17, 34, 49]. This was supported by findings showing that GPR40-null mice were protected against insulin resistance under high-fat diet [15]. In addition, two different GPR40 inhibitors were shown to rescue the deleterious effects of FFAs on insulin secretion and cell viability [17, 34]. In one study, Wu et al., also reported a small beneficial effect of GPR40 inhibition on palmitate-induced ER stress, contrary to our observations. It is currently unclear what underlies the opposing results of our study in comparison to those of others. However, differences in cell lines, palmitate concentration and the specific GPR40 antagonist molecule used might all play a role. Furthermore, the possibility for off-target effects of any small molecule antagonist cannot ever be fully excluded [17]. Furthermore, GPR40 antagonists have not been observed to promote better glucose levels or improvement in glucose tolerance in animal models [16, 49], which, therefore, suggest they offer little advantage as an antidiabetic drug.

In summary, we provide evidence that chronic activation of GPR40 does not lead to decreased cell viability and function or to increased oxidative and ER stresses. Nonetheless, our data do support an important role for GPR40 signaling in β-cell physiology, as demonstrated by the deleterious effects of the antagonist GW1100. Our data support the targeting of GPR40 for the treatment of T2DM and we believe that more efforts to test different agonists in clinical trials should be taken.

## Materials and methods

### Reagents

Culture medium with or without phenol red (RPMI 1640), dihydroethidium (DHE), RIPA lysis buffer, 3-(4,5-dimethylthiazol-2-yl)-2,5-diphenyltetrazolium bromide (MTT) and Fura-2 AM were obtained from Thermo Fischer Scientific (Waltham, MA, USA); palmitic acid was obtained from Sigma-Aldrich (St. Louis, MO, USA); GW9508 and GW1100 were obtained from Calbiochem/Merck Millipore (Billerica, MA, USA); ViaCount Reagent was obtained from Merck Millipore (Billerica, MA, USA); anti-p-eIF2-α (#9721S), anti-CHOP-10 (#2895S), anti-BiP (#3183), anti-α-tubulin (#3873) and anti-Ki67 (#12202) antibodies were obtained from Cell Signaling Technology (Danvers, MA, USA); Alexa Fluor555-conjugated anti-rabbit antibody (#A21429) was obtained from Invitrogen (Carlsbad, CA, USA).

### Stable expression of mito- or cyto-roGFP2-Orp1 in BRIN-BD11 cells

We have recently generated genetically encoded fluorescent protein redox sensors, which allow real-time measurements of defined redox species in intact living cells and animal models [50]. We are employing these cell lines in the context of a newly developed fluorescence plate-reader-based methodology that allows high-throughput screening of multiple different conditions. Herein, BRIN-BD11 cells expressing the genetically encoded H_2_O_2_ sensor roGFP2-Orp1 either in the mitochondrial matrix (Mito-roGFP2-Orp1 BRIN-BD11) or untargeted in the cytosol (roGFP2-Orp1 BRIN-BD11) were generated by lentivirus transduction. Briefly, HEK-293FT cells were transfected with pLPCX/mito-roGFP2-Orp1 or pLPCX/roGFP2-Orp1 (encoding the roGFP2-Orp1 probe with an N-terminal mitochondrial or non-targeted sequence) by calcium phosphate method. After 6 h of transfection, cells were washed twice with DMEM medium without supplements and transferred to fresh medium. After 48 h of transfection, supernatant was collected and passed through an Amicon column Spin-X 431,491 (Corning Inc., NY, USA). BRIN-BD11 cells (seeded at 3 × 10^5^ cells per well in a 6-well plate overnight before experiment) were incubated with the virus-containing supernatant in RPMI medium (0.1% P/S, 8 µg/ml Polybrene and without FCS) overnight at 37 °C in a 5% CO_2_ atmosphere. Cells were then selected in a medium containing puromycin (1 µg/ml) for 2 weeks.

### Culture and treatment of cell lines

BRIN-BD11 cells were grown in RPMI 1640 medium with 10% FCS, 0.1% P/S (100 U/ml penicillin, 0.1 mg/ml streptomycin) and 11.1 mmol/l D-glucose, pH 7.4, in a humidified atmosphere of 5% CO_2_/95% air at 37 °C. For flow cytometry assays (superoxide measurement and viability) and for western blot, cells were seeded at 6 × 10^4^ cells per well in 500-µl medium in 24-well plates; for the MTT assay and assessment of H_2_O_2_, cells were seeded at 7 × 10^3^ cells per well in 200-µl medium in 96-well plates; for the evaluation of proliferation by anti-Ki67 staining, cells were seeded at 2.5 × 10^3^ cells per condition in 500-µl medium in glass coverslips placed in 24-well plates; for the measurement of Ca^2+^ levels, cells were seeded at 1 × 10^4^ cells per condition in 1.5-ml medium in glass coverslips placed in 25 cm^2^ petri dishes. Mito-roGFP2-Orp1 BRIN-BD11 and roGFP2-Orp1 BRIN-BD11 cells were used for the assessment of H_2_O_2_ production. For the assessment of H_2_O_2_ and Ca^2+^, experiments were performed in complete RPMI medium without phenol red to decrease background fluorescence. Cells were allowed to adhere overnight and then incubated for 48 h in different conditions: 100 µM of PA, 20 µM of the GPR40 agonist GW9508, 10 µM of the GPR40 antagonist GW1100 or 100 µM of PA + 10 µM GW1100. All treatments were 48 h. PA was diluted in 100% ethanol to prepare a stock solution with a concentration of 50 mM. For complete solubilization, the stock solutions were mixed thoroughly. Stock solutions were prepared fresh for each experiment. GW1100 and GW9508 were diluted in DMSO to prepare stock solutions of 10 mM and 20 mM, respectively.

### Superoxide measurement

After 48 h exposure to treatment, cells were incubated with 40 µM of DHE dye for 20 min at room temperature. Cells were then washed with PBS and incubated with 100 µl of trypsin for 5 min at 37 °C. Trypsin was inactivated with 200 µl of complete RPMI medium and cells were transferred to a 96-well plate. Fluorescence was assessed by the flow cytometer Guava EasyCyte (Millipore). Values of each experiment were normalized by the respective control values (considered as 1).

### Hydrogen peroxide (H_2_O_2_) measurement

Measurements of intracellular levels of H_2_O_2_ were performed in mito-roGFP2-Orp1 BRIN-BD11 and roGFP2-Orp1 BRIN-BD11 cells after 48 h exposure to the different conditions, using the CLARIOstar Microplate Reader (BMG LABTECH, Ortenberg, Germany). Temperature was set to 37 °C and atmospheric conditions were set to 5% CO_2_ with the ventilation left open for O_2_ to diffuse freely in the system (18.8% O_2_). Cytosolic and mitochondrial roGFP emission was detected after excitation at 405 and 488 nm and emission at 500–530 nm. The ratio 405/488 nm was calculated and plotted against time using GraphPad Prism.

### Viability

Cell viability after 48 h of treatment was assessed by ViaCount Reagent, which distinguishes between viable and non-viable cells (divided into apoptotic and necrotic/dead cells). Protocol was performed as previously described [51] and fluorescence was assessed by the flow cytometer Guava EasyCyte (Millipore).

### Proliferation

The proliferation of the cells after 48 h of treatment was determined after immunostaining with anti-Ki67. After 48 h of incubation, the cells were fixed for 30 min in 4% paraformaldehyde (in PBS) and permeabilized for 10 min in PBS containing 0.2% Triton X-100 and 2% bovine serum albumin (BSA) at room temperature. Unspecific binding sites were blocked by incubation with 1% BSA in PBS for 15 min before cells were incubated overnight at 4 °C with the specific anti-Ki67 primary antibody. After washing with PBS, cells were incubated with secondary Alexa Fluor555-conjugated anti-rabbit antibody for 1 h at room temperature. Subsequently, the cells were washed and incubated for 15 min at 37 °C with 3.7% bisBenzimide solution to stain the nuclei and finally sealed with mounting media. Images were captured by fluorescence microscopy (BX60F; OlympusOptical, Tokyo, Japan) and quantified at ImageJ Fiji Software.

### MTT assay

The metabolic activity of cells after 48 h of treatment was determined using the MTT assay, as described by the manufacturer. Briefly, after addition of the MTT reagent to the samples, the purple formazan crystals formed were dissolved in DMSO and the absorbance was read at 570 nm on the microplate reader Synergy H1 (Biotek, Vermont, USA).

### Ca^2+^ measurements

For the analysis of cytosolic calcium after 48 h of treatment, BRIN-BD11 cells were loaded with 2-µM Fura-2 AM for 25 min in a bicarbonate-buffered Krebs solution and placed under the microscope Axio Observer 7 (Zeiss, Oberkochen, Germany). Measurements were made every 2 s for 15 min during two steps: first in buffer without glucose, followed by addition of high glucose (20 mM). Cells were imaged using excitation 340/380 nm and emission 505 nm.

### Western blot

Protein expression of ER stress markers was assessed by western blot. Total protein extracts were separated by SDS-PAGE and transferred to nitrocellulose membranes. Membranes were probed with specific antibodies against p-eIF2-α, CHOP and BiP. Anti-α-tubulin was used as internal control.

### Statistical analyses

Results are presented as mean ± SEM from at least three independent experiments. Statistical analysis was carried out at the GraphPad Prism seven software. Two-way ANOVA followed by Tukey was used to compare differences between the following groups: control, PA, GW1100 and PA + GW1100; Student’s *t* test was used to compare differences between control and GW9508. In the supplementary Fig. 2 , one-way ANOVA followed by Dunnet was used. Confidence levels were set to *p* < 0.05. *P* values, *F* values and degree of freedom for the two-way ANOVA (column factor and row factor) are presented in the figure legends. *P* values for the Tukey post test or Student’s t-test are presented in the figures.

## Electronic supplementary material

Below is the link to the electronic supplementary material.

Supplementary file1 (PDF 374 kb)

## References

[1] Graciano MF, Valle MM, Kowluru A, Curi R, Carpinelli AR (2011). Regulation of insulin secretion and reactive oxygen species production by free fatty acids in pancreatic islets. Islets..

[2] Graciano MF, Valle MM, Curi R, Carpinelli AR (2013). Evidence for the involvement of GPR40 and NADPH oxidase in palmitic acid-induced superoxide production and insulin secretion. Islets..

[3] Nolan CJ, Madiraju MS, Delghingaro-Augusto V, Peyot ML, Prentki M (2006). Fatty acid signaling in the beta-cell and insulin secretion. Diabetes.

[4] Poitout V (2003). The ins and outs of fatty acids on the pancreatic beta cell. Trends Endocrinol Metab..

[5] El-Assaad W, Buteau J, Peyot ML, Nolan C, Roduit R, Hardy S (2003). Saturated fatty acids synergize with elevated glucose to cause pancreatic beta-cell death. Endocrinology.

[6] Karaskov E, Scott C, Zhang L, Teodoro T, Ravazzola M, Volchuk A (2006). Chronic palmitate but not oleate exposure induces endoplasmic reticulum stress, which may contribute to INS-1 pancreatic beta-cell apoptosis. Endocrinology.

[7] Lai E, Bikopoulos G, Wheeler MB, Rozakis-Adcock M, Volchuk A (2008). Differential activation of ER stress and apoptosis in response to chronically elevated free fatty acids in pancreatic beta-cells. Am J Physiol Endocrinol Metab..

[8] Cunha DA, Hekerman P, Ladrière L, Bazarra-Castro A, Ortis F, Wakeham MC (2008). Initiation and execution of lipotoxic ER stress in pancreatic beta-cells. J Cell Sci..

[9] Cnop M, Ladrière L, Igoillo-Esteve M, Moura RF, Cunha DA (2010). Causes and cures for endoplasmic reticulum stress in lipotoxic β-cell dysfunction. Diabetes Obes Metab..

[10] Gehrmann W, Elsner M, Lenzen S (2010). Role of metabolically generated reactive oxygen species for lipotoxicity in pancreatic β-cells. Diabetes Obes Metab..

[11] Maris M, Robert S, Waelkens E, Derua R, Hernangomez MH, D'Hertog W (2013). Role of the saturated nonesterified Fatty Acid palmitate in Beta cell dysfunction. J Proteome Res..

[12] Gehrmann W, Würdemann W, Plötz T, Jörns A, Lenzen S, Elsner M (2015). Antagonism Between Saturated and Unsaturated Fatty Acids in ROS Mediated Lipotoxicity in Rat Insulin-Producing Cells. Cell Physiol Biochem..

[13] Michalska M, Wolf G, Walther R, Newsholme P (2010). Effects of pharmacological inhibition of NADPH oxidase or iNOS on pro-inflammatory cytokine, palmitic acid or H2O2-induced mouse islet or clonal pancreatic β-cell dysfunction. Biosci Rep..

[14] Wei Y, Wang D, Topczewski F, Pagliassotti MJ (2006). Saturated fatty acids induce endoplasmic reticulum stress and apoptosis independently of ceramide in liver cells. Am J Physiol Endocrinol Metab..

[15] Steneberg P, Rubins N, Bartoov-Shifman R, Walker MD, Edlund H (2005). The FFA receptor GPR40 links hyperinsulinemia, hepatic steatosis, and impaired glucose homeostasis in mouse. Cell Metab..

[16] Zhang X, Yan G, Li Y, Zhu W, Wang H (2010). DC260126, a small-molecule antagonist of GPR40, improves insulin tolerance but not glucose tolerance in obese Zucker rats. Biomed Pharmacother..

[17] Wu J, Sun P, Zhang X, Liu H, Jiang H, Zhu W (2012). Inhibition of GPR40 protects MIN6 β cells from palmitate-induced ER stress and apoptosis. J Cell Biochem..

[18] Itoh Y, Hinuma S (2005). GPR40, a free fatty acid receptor on pancreatic beta cells, regulates insulin secretion. Hepatol Res..

[19] Briscoe CP, Tadayyon M, Andrews JL, Benson WG, Chambers JK, Eilert MM (2003). The orphan G protein-coupled receptor GPR40 is activated by medium and long chain fatty acids. J Biol Chem..

[20] Shapiro H, Shachar S, Sekler I, Hershfinkel M, Walker MD (2005). Role of GPR40 in fatty acid action on the beta cell line INS-1E. Biochem Biophys Res Commun..

[21] Itoh Y, Kawamata Y, Harada M, Kobayashi M, Fujii R, Fukusumi S (2003). Free fatty acids regulate insulin secretion from pancreatic beta cells through GPR40. Nature.

[22] Fujiwara K, Maekawa F, Yada T (2005). Oleic acid interacts with GPR40 to induce Ca2+ signaling in rat islet beta-cells: mediation by PLC and L-type Ca2+ channel and link to insulin release. Am J Physiol Endocrinol Metab..

[23] Negoro N, Sasaki S, Mikami S, Ito M, Suzuki M, Tsujihata Y (2010). Discovery of TAK-875: A Potent, Selective, and Orally bioavailable GPR40 Agonist. ACS Med Chem Lett..

[24] Houze JB, Zhu L, Sun Y, Akerman M, Qiu W, Zhang AJ (2012). AMG 837: a potent, orally bioavailable GPR40 agonist. Bioorg Med Chem Lett.

[25] Araki T, Hirayama M, Hiroi S, Kaku K (2012). GPR40-induced insulin secretion by the novel agonist TAK-875: first clinical findings in patients with type 2 diabetes. Diabetes Obes Metab..

[26] Mohammad S (2016). GPR40 Agonists for the treatment of type 2 diabetes mellitus: benefits and challenges. Curr Drug Targets..

[27] Mancini AD, Poitout V (2013). The fatty acid receptor FFA1/GPR40 a decade later: how much do we know?. Trends Endocrinol Metab..

[28] Mancini AD, Poitout V (2015). GPR40 agonists for the treatment of type 2 diabetes: life after 'TAKing' a hit. Diabetes Obes Metab..

[29] Naik H, Vakilynejad M, Wu J, Viswanathan P, Dote N, Higuchi T (2012). Safety, tolerability, pharmacokinetics, and pharmacodynamic properties of the GPR40 agonist TAK-875: results from a double-blind, placebo-controlled single oral dose rising study in healthy volunteers. J Clin Pharmacol..

[30] Burant CF, Viswanathan P, Marcinak J, Cao C, Vakilynejad M, Xie B (2012). TAK-875 versus placebo or glimepiride in type 2 diabetes mellitus: a phase 2, randomised, double-blind, placebo-controlled trial. Lancet.

[31] Leifke E, Naik H, Wu J, Viswanathan P, Demanno D, Kipnes M (2012). A multiple-ascending-dose study to evaluate safety, pharmacokinetics, and pharmacodynamics of a novel GPR40 agonist, TAK-875, in subjects with type 2 diabetes. Clin Pharmacol Ther..

[32] Kaku K, Araki T, Yoshinaka R (2013). Randomized, double-blind, dose-ranging study of TAK-875, a novel GPR40 agonist, in Japanese patients with inadequately controlled type 2 diabetes. Diabetes Care.

[33] Meidute Abaraviciene S, Lundquist I, Galvanovskis J, Flodgren E, Olde B, Salehi A (2008). Palmitate-induced beta-cell dysfunction is associated with excessive NO production and is reversed by thiazolidinedione-mediated inhibition of GPR40 transduction mechanisms. PLoS ONE.

[34] Kristinsson H, Smith DM, Bergsten P, Sargsyan E (2013). FFAR1 is involved in both the acute and chronic effects of palmitate on insulin secretion. Endocrinology.

[35] Natalicchio A, Labarbuta R, Tortosa F, Biondi G, Marrano N, Peschechera A (2013). Exendin-4 protects pancreatic beta cells from palmitate-induced apoptosis by interfering with GPR40 and the MKK4/7 stress kinase signalling pathway. Diabetologia.

[36] Menon V, Lincoff AM, Nicholls SJ, Jasper S, Wolski K, McGuire DK (2018). Fasiglifam-induced liver injury in patients with type 2 diabetes: results of a randomized controlled cardiovascular outcomes safety trial. Diabetes Care.

[37] Roma LP, Deponte M, Riemer J, Morgan B (2018). Mechanisms and applications of redox-sensitive green fluorescent protein-based hydrogen peroxide probes. Antioxid Redox Signal..

[38] Schwarzländer M, Dick TP, Meyer AJ, Morgan B (2016). Dissecting redox biology using fluorescent protein sensors. Antioxid Redox Signal..

[39] Morgan D, Oliveira-Emilio HR, Keane D, Hirata AE, Santos da Rocha M, Bordin S (2007). Glucose, palmitate and pro-inflammatory cytokines modulate production and activity of a phagocyte-like NADPH oxidase in rat pancreatic islets and a clonal beta cell line. Diabetologia.

[40] Carlsson C, Borg LA, Welsh N (1999). Sodium palmitate induces partial mitochondrial uncoupling and reactive oxygen species in rat pancreatic islets in vitro. Endocrinology.

[41] Barlow J, Affourtit C (2013). Novel insights into pancreatic β-cell glucolipotoxicity from real-time functional analysis of mitochondrial energy metabolism in INS-1E insulinoma cells. Biochem J..

[42] Sato Y, Fujimoto S, Mukai E, Sato H, Tahara Y, Ogura K (2014). Palmitate induces reactive oxygen species production and β-cell dysfunction by activating nicotinamide adenine dinucleotide phosphate oxidase through Src signaling. J Diabetes Investig..

[43] Fu J, Cui Q, Yang B, Hou Y, Wang H, Xu Y (2017). The impairment of glucose-stimulated insulin secretion in pancreatic β-cells caused by prolonged glucotoxicity and lipotoxicity is associated with elevated adaptive antioxidant response. Food Chem Toxicol..

[44] Shen X, Yang L, Yan S, Wei W, Liang L, Zheng H (2014). The effect of FFAR1 on pioglitazone-mediated attenuation of palmitic acid-induced oxidative stress and apoptosis in βTC6 cells. Metabolism..

[45] Wagner R, Kaiser G, Gerst F, Christiansen E, Due-Hansen ME, Grundmann M (2013). Reevaluation of fatty acid receptor 1 as a drug target for the stimulation of insulin secretion in humans. Diabetes.

[46] Deglasse JP, Roma LP, Pastor-Flores D, Gilon P, Dick TP, Jonas JC (2019). Glucose acutely reduces cytosolic and mitochondrial H2O2 in rat pancreatic beta cells. Antioxid Redox Signal.

[47] Takahashi HK, Santos LR, Roma LP, Duprez J, Broca C, Wojtusciszyn A (2014). Acute nutrient regulation of the mitochondrial glutathione redox state in pancreatic β-cells. Biochem J..

[48] Kristinsson H, Bergsten P, Sargsyan E (2015). Free fatty acid receptor 1 (FFAR1/GPR40) signaling affects insulin secretion by enhancing mitochondrial respiration during palmitate exposure. Biochim Biophys Acta..

[49] Sun P, Wang T, Zhou Y, Liu H, Jiang H, Zhu W (2013). DC260126: a small-molecule antagonist of GPR40 that protects against pancreatic β-Cells dysfunction in db/db mice. PLoS ONE.

[50] Fujikawa Y, Roma LP, Sobotta MC, Rose AJ, Diaz MB, Locatelli G (2016). Mouse redox histology using genetically encoded probes. Sci Signal..

[51] Lucena CF, Roma LP, Graciano MF, Veras K, Simões D, Curi R (2015). Omega-3 supplementation improves pancreatic islet redox status: in vivo and in vitro studies. Pancreas.

