# Supplementary figures and images for: Chronic activation of GPR40 does not negatively impact upon BRIN-BD11 pancreatic β-cell physiology and function
Source: Pharmacol Rep. 2020 Apr 9;72(6):1725–37. doi: 10.1007/s43440-020-00101-6 (PMC7704488; doi:10.1007/s43440-020-00101-6)

# Supplementary figure 1

**A**

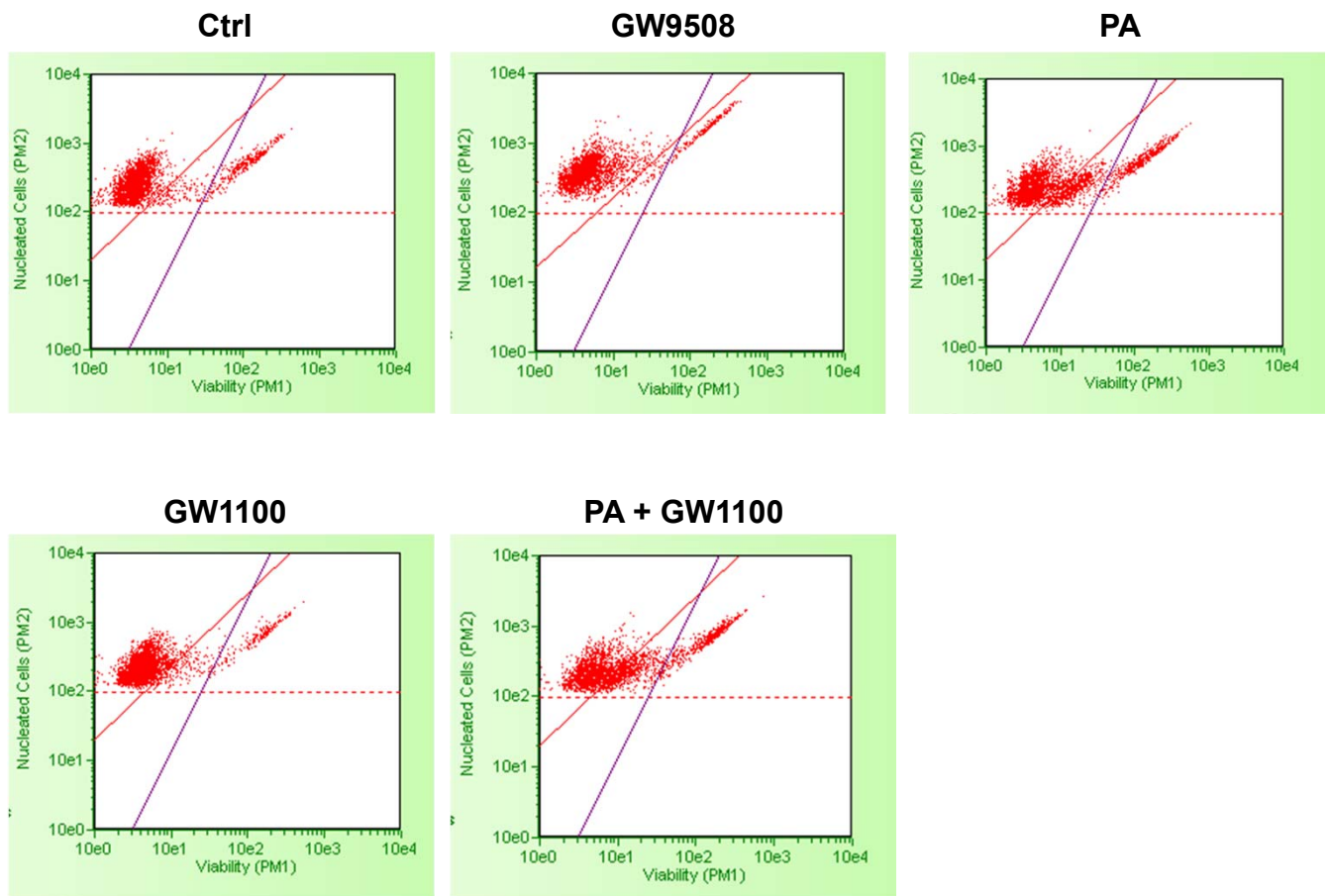

## Supplementary figure 2

**A**

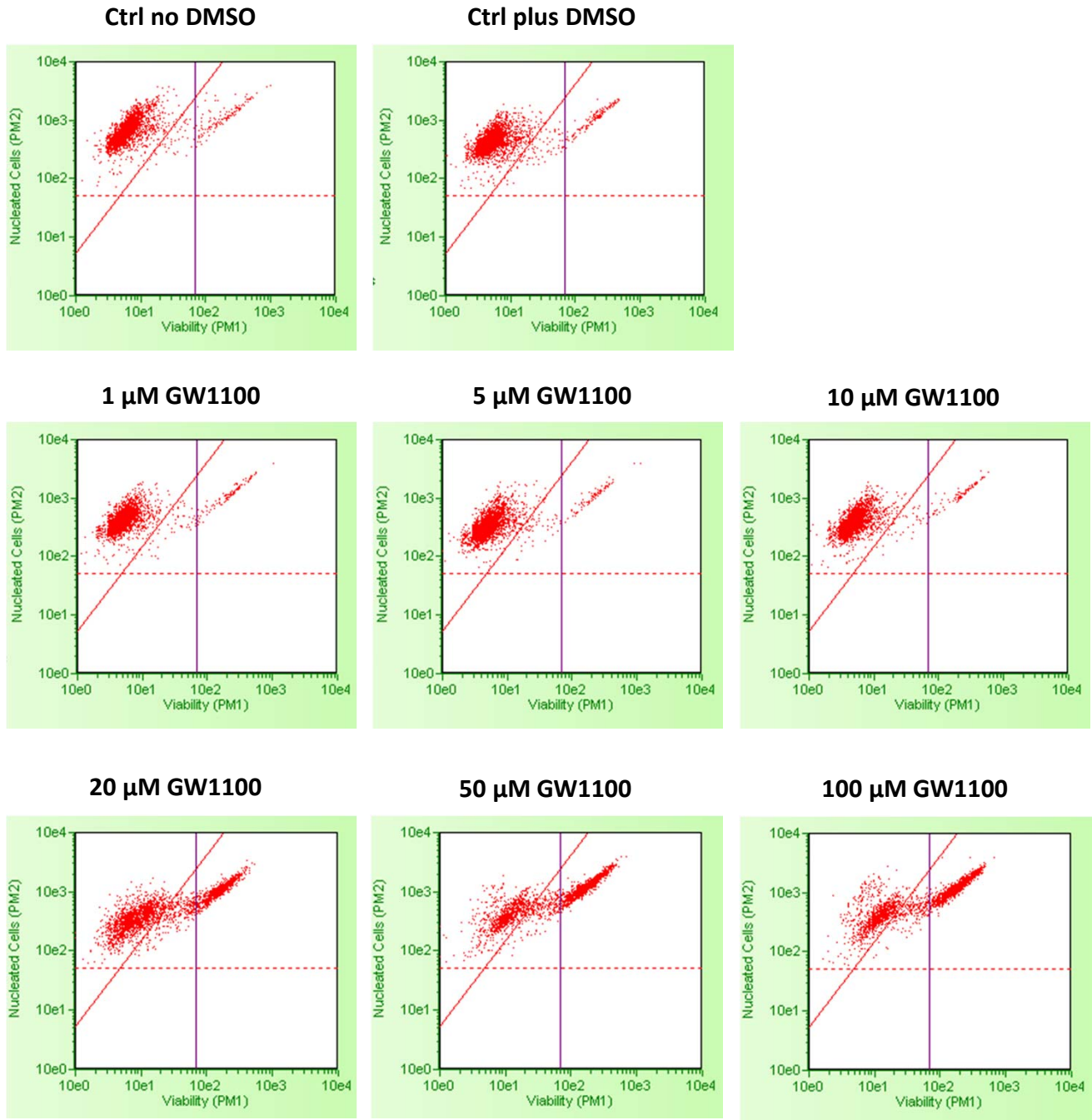

**B**

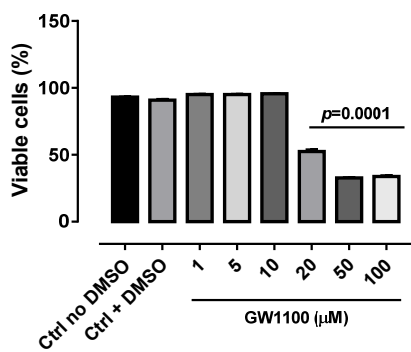

**C**

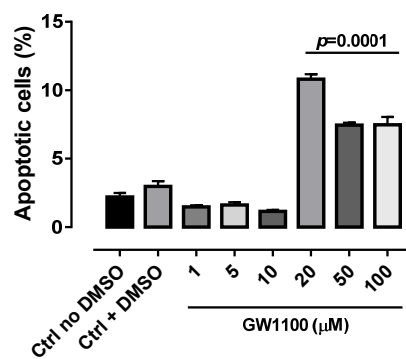

**D**

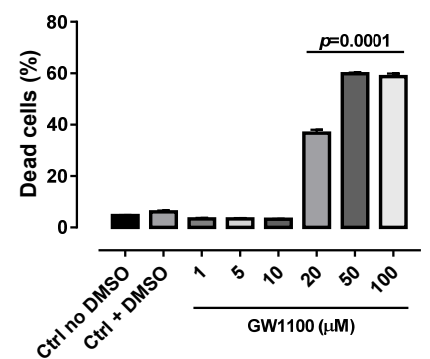

Supplement: Supplementary file 1 — Supplementary file1 (PDF 374 kb) [file 43440_2020_101_MOESM1_ESM.pdf]
